# A Novel Evaluation of Income Class Boundaries Using Inflection Points of Probability Density Functions: A Case Study of Brazil

**DOI:** 10.3390/e27020186

**Published:** 2025-02-12

**Authors:** Rafael Bittencourt, Hernane Borges de Barros Pereira, Marcelo A. Moret, Ivan C. Da Cunha Lima, Serge Galam

**Affiliations:** 1 Modelagem Computacional, Universidade SENAI CIMATEC, Salvador 41650-010, Bahia, Brazil; almeidabittencourt@gmail.com (R.B.); hernanebbpereira@gmail.com (H.B.d.B.P.); mamoret@gmail.com (M.A.M.); ivandacunhalima@gmail.com (I.C.D.C.L.); 2 Instituto Nacional de Ciência e Tecnologia—Geofísica do Petróleo, Salvador 41650-010, Bahia, Brazil; 3 Department of Education, Bahia State University, Salvador 41150-000, Bahia, Brazil; 4 CEVIPOF—Centre for Political Research, Sciences Po and CNRS, 75007 Paris, France

**Keywords:** complex systems, social phenomena, statistics, inflection points, metalog distributions, Shannon entropy, Gini coefficients, income classes

## Abstract

Categorizing a population into different income classes is important for creating effective policies and analyzing markets. Our study develops a statistical method based on a nationwide survey of income distribution. We use these data to create a cumulative distribution function with a metalogistic distribution and its probability density function. We propose a new way to divide the population into income classes by using the inflection points of the probability density function as the class boundaries. As a case study, we apply this method to income data from Brazil between 2012 and 2022. We identify five income classes, with both their boundaries and the distribution of the population changing over time. To check our approach, we calculate the Gini coefficient and find that our results closely match official figures, with a root mean square deviation of less than 1%. By using individual income instead of family income, we avoid distortions caused by the fact that poorer families tend to be larger than wealthier ones. In the end, we identify five main income classes, with their boundaries shifting each year, reflecting the changing nature of income distribution in society.

## 1. Introduction

Human societies classify groups based on various criteria, including caste, ethnicity, or a combination of origin and phenotype. When it comes to economic status, classification typically divides people into lower, middle, and upper classes. The lower class consists of those struggling for basic survival, while the upper class includes individuals with substantial financial resources, ranging from comfortable and stable lives to those with wealth beyond measure. The middle class falls between these two extremes. The boundaries between these classes are often arbitrary and can vary from country to country.

Classifying individuals into these categories helps shape strategic state policies and marketing strategies. Understanding the characteristics of each class is crucial for both applications. Artificial intelligence is increasingly effective in identifying these characteristics and preferences. Over time, individuals may move within their original class or transition to a neighboring class, reflecting changes in their economic status and social mobility.

Income distributions have been studied in many countries [1]. A study of the income distribution in the USA [2,3,4] with data provided by the the Internal Revenue Service (IRS) and from the US Census Bureau, found that income distribution is exponential for incomes below USD 120 k per year and that the upper tail of income distribution follows a power law [5] This two-class model was used to analyze the temporal evolution of the income classes in the USA during 1983–2001 [6] and, more recently, during 1983–2018 [7].

A new methodology [8], based on the two-class model, was developed to estimate income distribution while accounting for hidden sources of income, such as retained earnings. This approach was applied in a case study of the Chilean economy.

Aiming to adapt the National Innovation System (NIS), a mechanism to provide means to public decision-makers on issues of innovation, knowledge, and economic development to low- and middle-income countries, a proposal [9] of a qualitative model was validated in the case of Senegal. A low-income economy, it draws most of its efforts not in terms of S&T but, more broadly, in its learning policy, its reforms on higher education, or proposals on entrepreneurship.

An analysis of personal income distribution in Australia from 1899 to 2000 involved fitting exponential, log-normal, and gamma distributions to data provided by the Australian Bureau of Statistics [10]. The survey included 14,000 individuals from a total population of 20 million in Australia.

A large study on social stratification, which assumes that a society’s wealth is linked to the energy available from prehistoric times to today [11], suggests that the dynamics of social stratification can be seen as a stochastic process following the principle of maximum entropy [12,13]. The study presents an entropic perspective on wealth.

Much of the analysis made on measuring the inequality of income in a population uses the Gini coefficient [14,15,16,17,18]. This criterion considers the total income of the population and how it is distributed. Other criteria are also used, like income tax data and deciles of mean incomes. But until now, no agreed-upon criteria have been established, aside from the challenge of obtaining accurate income measures. Yet, it is of central importance to obtain a reliable representation of the distribution of income to design appropriate new public policies to correct the existing social inequalities.

Here, we propose an alternative criterion for identifying the existing distinct classes according to their respective mean incomes. This criterion comes in addition to the other ones to shed a different light on existing distinct classes and, in particular, to allow a better shaping of public policies. Our approach relates to specific properties of the cumulative probability function of the individual income distribution of the population. Our hypothesis is to associate a change in the slope of the probability density function of monthly income *per capita* to a change of social category. Having the associated repartition in classes allows us to study the crossings between classes boundaries from one year to the next one. This scheme provides a novel tool to evaluate the efficiency of applied public policies. As a case study, we applied our criterion to identify income classes in Brazil. Our results show the existence of five classes, whose boundaries and population change with time. We analyzed those changes in a period of eleven years, from 2012 to 2022, covering several presidential terms.

This work subscribes to the field of physics-like modeling of social, economics, and financial phenomena [19,20,21,22,23,24,25].

The remainder of the paper is organized as follows: Section 2 outlines the criterion for defining class boundaries, describes the use of the metalogistic distribution to fit the cumulative probability function, and provides a preliminary discussion of how Brazilian society is typically categorized using annual surveys conducted by the Brazilian government. In Section 3, we apply our criterion to the *per capita* monthly income distribution data from Brazilian agencies, covering the period from 2012 to 2022 across four presidential terms. To validate our quantile probability function, we compute the Gini coefficients for this period. Section 4 analyzes our results, examining the flow between classes and changes in their boundaries. Finally, Section 5 highlights the advantages of our approach and compares our classifications with those of the Brazilian statistical agency.

## 2. Materials and Methods

### 2.1. The Metalog Distribution Method

Let *x* represent the observable data used to measure the individual’s position in the social scale, such as individual income. We define the cumulative probability function (CPF) as y=F(x), where F(x) is the fraction of the population with values below *x*. Thus the fraction of the population between $x_{1}$ and $x_{2}$ (where $x_{2}>x_{1}$) is given by $F\left(x_{2}\right)-F\left(x_{1}\right)$. The cumulative probability function satisfies F(0)=0 and $F(x_{\max})=1$, where $x_{\max}$ is the maximum observed value within the group.

The quantile probability function (QPF), denoted as Q(y), is defined as the inverse function of the cumulative probability function F(x). It provides the value of the observable data corresponding to a given fraction (*y*) of the population, such that y=F(x), x=Q(y).

For a tiny interval dx corresponding to a tiny interval dy, there exists a function f(x) that maps one interval into the other: dy=f(x)dx. This function f(x) is the probability density function (PDF) and represents the likelihood of finding individuals within the interval between *x* and x+dx:

(1) $$f\left(x\right)=\frac{dy}{dx}={\left(\frac{dx}{dy}\right)}^{-1}={\left(\frac{dQ(y)}{dy}\right)}^{-1}.$$

Therefore, as indicated by Equation (1), once the quantile probability function is derived from the survey data, its derivative yields the probability density function of the distribution.

A sigmoidal distribution with a cumulative probability function y=F(x) is associated with bell-shaped probability density function (PDF), denoted as f(x). We refer to this PDF as f(x;μ,s). The function f(x;μ,s) describes the distribution of the observable (variable *x*) around x=μ with a spread, *s*. The probability *y* of observing a value of $x^{\prime }\leq x$ is

(2) $$y=F\left(x;\mu ,s\right)=\int_{0}^{x}f\left(x^{\prime };\mu ,s\right)dx^{\prime }$$

Similarly, the probability density function (PDF) for the distribution of variable *y* is the derivative of the quantile probability function (QPF) given in Equation (1), which is also known as the quantile density function (q-PDF).

In this study, we employed the sigmoidal metalogistic probability function (metalog distribution) [26] due to its flexibility in shaping the distribution. Introduced in 2016, the metalog distribution has been widely applied across various fields. It extends the logistic distribution, offering enhanced adaptability for modeling diverse data patterns:

(3) $$Q\left(y\right)=\mu +s\ln\frac{y}{1-y},$$

with a power series expansion for location (μ) and scale parameters (*s*):

(4) $$\begin{array}{ccc} \mu & = & a_{1}+a_{4}\left(y-0.5\right)+a_{5}{\left(y-0.5\right)}^{2}+a_{7}{\left(y-0.5\right)}^{3}+a_{9}{\left(y-0.5\right)}^{4}+\ldots \\ s & = & a_{2}+a_{3}\left(y-0.5\right)+a_{6}{\left(y-0.5\right)}^{2}+a_{8}{\left(y-0.5\right)}^{3}+a_{10}{\left(y-0.5\right)}^{4}+\ldots \end{array}$$

The expansions in Equation (4) use the property that, for a sigmoidal distribution with a cumulative probability function y=F(x), the function *y* is approximately linear in *x* within a small neighborhood around x=μ. Alternatively, if we treat μ and *s* as functions of *y*, they can be expressed as power series expansions around fixed values $\mu =a_{1}$ and $s=a_{2}$. This approach serves as the foundation for defining the metalogistic distribution. Note that Equation (4) do not represent a polynomial expansion of the cumulative probability function itself; rather, they describe expansions for the location and scale parameters of the quantile probability function. The fitting parameters $a_{1},a_{2}\ldots$ are utilized in the expansions for μ and *s*. We present these expansions in the following traditional sequence: the first parameter is for μ, the second and third parameters are for *s*, the fourth and fifth parameters are for μ, and from the sixth parameter onward, they alternate between μ and *s*, continuing up to the *k*-th parameter.

We hypothesize that class boundaries are likely indicated by changes in the trend of the probability density function (PDF), either increasing or decreasing. It is natural to associate these boundaries with inflection points in the PDF, where the second derivative of the PDF is zero.

Associating class boundaries with the zeros of the second derivative of the probability density function (PDF) evokes concepts from the Lee–Yang theory [27,28], which is used to describe phase transitions in the thermodynamic limit. In this context, the number of particles and the system volume approach infinity while the particle density remains finite. A phase transition occurs when there is a sudden change—whether continuous or discontinuous—in a system property. The Lee–Yang theory links phase transitions to the zeros of the partition function in the complex plane [29,30].

The PDF is relevant primarily for very dense systems or in the thermodynamic limit of finite systems. The zeros of the second derivative of the PDF indicate sudden changes—whether continuous or discontinuous—in the characteristics of classes, thereby establishing the boundaries of these classes on the social scale. The Lee–Yang theory has broad applications in various fields, including protein folding, complex networks, and percolation [31,32,33,34,35,36,37,38,39].

### 2.2. Case Study: Analyzing Brazilian Income Distribution

As a case study, we examine the *per capita* income distribution in Brazil, a country with a long history of affirmative action policies. These policies include financial support for low-income families, preferential admission to public universities, access to public services, and eligibility for political party candidacies, among other areas. Brazil has had a minimum income policy for over three decades, with its values and designations varying according to the ruling party.

In Brazil, social classes are commonly categorized into three broad groups based on family income: upper class, middle class, and lower class. However, the economic classification system employed by the Secretariat of Strategic Affairs (SAE) and the Brazilian Association of Research Companies (ABEP) provides a more detailed breakdown. This system further divides these broad categories into more specific classifications, denoted by letters. These classifications are the following:

- Class A: A1, A2.
- Class B: B1, B2.
- Class C: C1, C2.
- Class D.
- Class E.

Among these classifications, Class A1 represents the highest economic status, characterized by superior quality of life and greater purchasing power. In contrast, Class E signifies the lowest economic status, with lower purchasing power and reduced quality of life. This classification takes into account factors such as family income, assets, and education levels. However, our study focuses solely on income values, excluding other social parameters.

For consistency, we use the Brazilian national currency as the unit of *per capita* income throughout this work. As of 2024, the minimum wage in Brazil is BRL 1412 (approximately USD 300). However, given the significant presence of the informal labor market in Brazil, the minimum wage may not fully reflect the economic reality for many individuals.

The Brazilian Institute of Geography and Statistics (IBGE) categorizes social classes based on monthly family income into five main groups:

- Class A: Above 20 minimum wages (≥BRL 28,240).
- Class B: From 10 to 20 minimum wages (BRL 14,120–BRL 28,240).
- Class C: From 4 to 10 minimum wages (BRL 5648–BRL 14,120).
- Class D: From 2 to 4 minimum wages (BRL 2824–BRL 5648).
- Class E: Up to 2 minimum wages (≤BRL 2824).

The classifications mentioned are purely economic and static, focusing on family incomes. It is known that the broad category of the lower class generally includes families with a larger number of members compared to those in the middle and upper classes. In contrast, our criterion focuses on individual income, allowing a dynamic analysis of class boundaries. This approach enables us to examine the movement of individuals between classes and provides a more nuanced understanding of social mobility.

Our case study utilizes data from the Brazilian Institute of Geography and Statistics (IBGE), the Brazilian agency responsible for collecting and analyzing data to inform governmental strategies [40]. Table 1 presents the *per capita* income distribution derived from eleven consecutive surveys conducted between 2012 and 2022. The data are categorized by IBGE into twelve percentiles, each representing 10% of the population, with the exception of the final two percentiles, which cover the 90–95% and 95–99% ranges. The values in the columns represent the highest income within each percentile slice.

The top 1% is not included in the table, as the income distribution in this segment spans a broad range of values, starting from those listed in the last row of Table 1. This omission would complete the twelve slices of Brazil’s income distribution. It is important to note that the table shows the upper *per capita* income for fixed population fractions. Hence, if we overlook the gradual increase in population over the eleven-year period, the number of individuals in each percentile slice remains constant over time.

Table 1 clearly illustrates a striking disparity in income distribution among the population. The extent of this income dispersion is so significant that we had to use a logarithmic scale to ensure a more accurate statistical analysis. Despite Brazil experiencing several decades without major natural or economic disasters, and remaining unaffected by war or sudden political upheavals, the income inequality has persisted. This inequality has endured even as Brazil maintained its position among the world’s top ten largest economies.

Low-income families in Brazil are estimated to number around 30 million out of a total population of approximately 220 million. These families receive support from various social programs provided by the Brazilian government, including the Bolsa Família program, which offers BRL 600 per family, subject to certain conditions. Additional benefits are provided for each child in the family.

Brazil, a Federative Republic, holds presidential elections every four years. The election prior to 2012 was held in 2010, covering the presidential term from 2011 to 2015. The data presented in Table 1 span from 2012 to 2022, encompassing four presidential terms: 2011–2014 under Luiz Inácio Lula da Silva, 2015–2018 under Dilma Rousseff and Michel Temer, and 2019–2022 under Jair Messias Bolsonaro.

## 3. Results

### 3.1. The Metalogistic Distribution

The statistical methodology is explained in detail in Section 2.1. The first step in our analysis involves obtaining the quantile probability function (QPF) by inverting the data in Table 1, where y=F(x) and x=Q(y) for y=10,20,…, along with the corresponding upper income values for each year. This step results in a list of Q(y) values for the eleven selected percentiles, which are then used to fit the metalog quantile function.

We fitted the quantile function with trial metalog functions for values of *k* ranging from 5 to 10. Table 2 displays the coefficients of the metalog distribution.

We employed the mean absolute error (MAE) as the evaluation metric, rather than the mean squared error (MSE). While MSE penalizes larger errors more severely by squaring the differences, MAE treats all errors equally by averaging the absolute differences. This characteristic makes MAE more robust, particularly in datasets where outliers might disproportionately influence the results.

**Figure 1:**
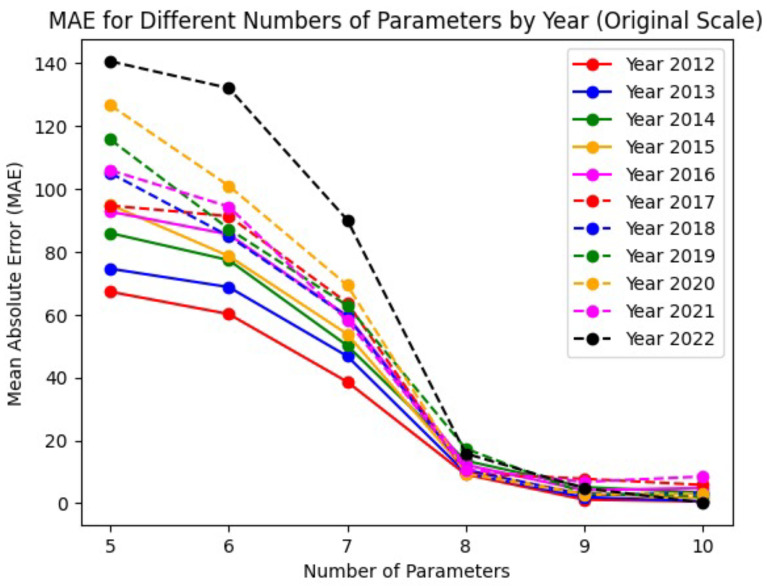
Mean absolute error (MAE) for each year from 2012 to 2022, for metalog quantile functions with different numbers of parameters (from k=5 up to k=10).

**Figure 2:**
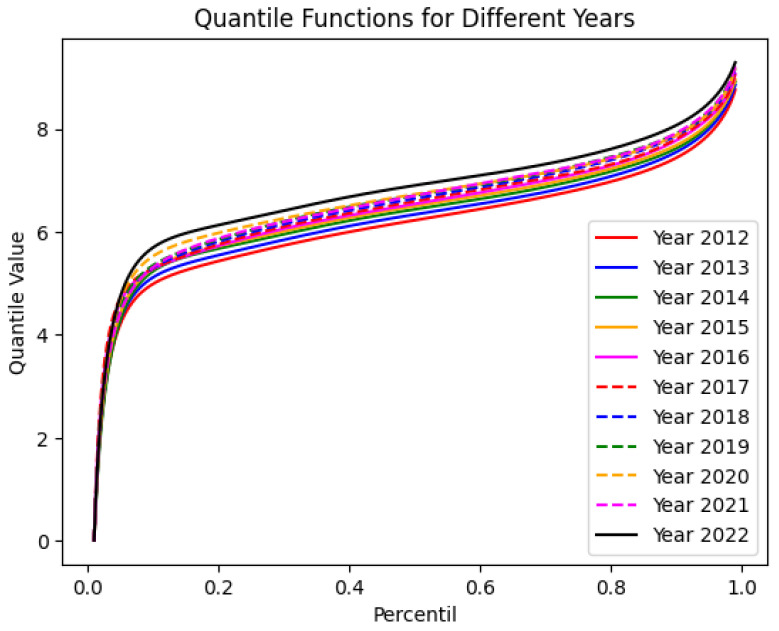
Quantile probability function of the logarithm of monthly *per capita* income for the population segments in Table 1. The curves are derived from fitting a metalogistic distribution function with k=10.

Figure 1 presents the MAE for each year from 2012 to 2022, comparing metalog quantile functions with different numbers of parameters ranging from k=5 to k=10. Each line in the plot represents a different year, with colors and line styles corresponding to those used in the quantile function plots. This visualization facilitates a detailed assessment of model performance across various years, allowing for the identification of specific patterns or anomalies. For our analysis, we chose k=10 in the statistical treatment. It is important to note that *k* represents the number of parameters used in the expansions of μ and *s*, not the degree of a polynomial fitting the PDF F(x). The plots in Figure 2 illustrate the quantile probability functions (QPFs) with a characteristic sigmoidal shape over the eleven-year period.

### 3.2. The Shannon Entropy and the Gini Coefficients

Any closed macroscopic system when departed from its equilibrium and left alone tends to recover its equilibrium. In non-equilibrium statistical mechanics, entropy, S(t), is a function of the probability density that describes the state of a system with weak interactions, governed by a kinetic equation [13]. This function is well defined and always increases over time towards the equilibrium, as demonstrated by the Boltzmann H-theorem. Does the wealth of a human society evolve over a large timescale to an equilibrium? Does this equilibrium, if existing, support stratification? A profound discussion on the misinterpretation of the term “entropy” in social sciences [12] concludes the income distribution data for USA and Sweden to be consistent with the principle of maximum entropy when used within the Pareto distribution. A deep analysis of stratification of a society using an entropic approach [11] treated the energy used of humans in a hunter–gatherer society represented by a stochastic variable *x* and a probability density function f(x). The authors define the income entropy as

(5) $$\Phi =-\int_{0}^{I}f\left(x\right)\ln f\left(x\right)dx,$$

where *I* is th upper value of the random variable *x*.

On the other hand, a widely used definition of “entropy” in complex systems is the Shannon entropy, given by

(6) $$S=\ln\sigma \sqrt{2\pi e},$$

where σ is the standard deviation of the distribution, corresponding to the second moment of the probability density function. Table 3 shows the calculated values of the Shannon entropy for each year in the period 2012–2022. At this stage, a word of caution is in order here. Indeed, there is no evidence that the income data we used represent a stochastic process. The system is not isolated and, thus, does not tend towards equilibrium. Therefore, Boltzmann’s H-theorem does not apply. Moreover, Shannon’s entropy only serves to renormalize the width of the distribution. Yet, it could be a measure of how inequalities increase when they increase.

To ensure completeness, we calculated the Gini coefficients using the data in Table 1. This calculation also serves as a validation criterion for our quantile probability function, which is used to determine the boundaries between classes.

**Figure 3:**
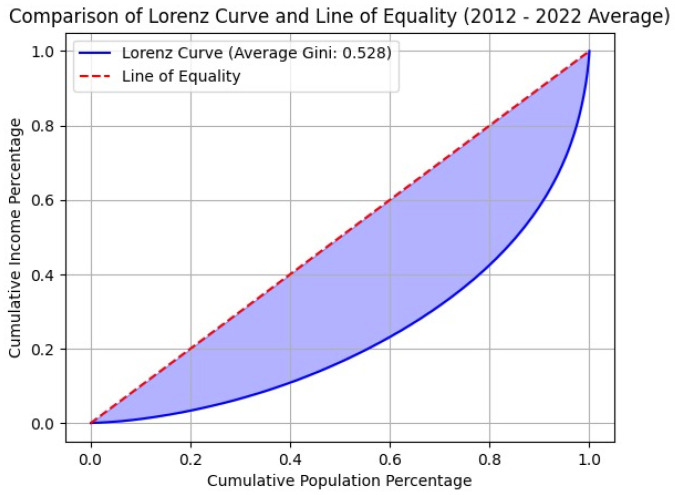
The figure presents the average Lorenz curve derived from the metalogistic treatment with k=10 for the period 2012–2022. The Gini coefficient associated with this curve is G=0.528.

The Gini coefficient [14] is a well-established measure of income inequality within a society. Figure 3 illustrates the Lorenz curve, which shows the proportion of total income earned by a given fraction of the population relative to that fraction. In a perfectly equal society, the Lorenz curve would be represented by a straight line, L(x)=x. The Gini coefficient is calculated as the area between the actual Lorenz curve and the line of perfect equality (depicted in blue), divided by the area under the line of perfect equality. A larger Gini coefficient signifies greater income inequality.

In the plot, the horizontal axis represents the variable *y* used in this work, while the vertical axis shows the cumulative income percentage of the population, denoted as variable *z*. The cumulative income is yet to be calculated. Our analysis provides the cumulative probability function y=F(x), which gives the percentage of the population earning up to a given individual income *x*. Additionally, the inverse function Q(y), or the quantile probability function, provides the maximum individual income for a given fraction *y* of the population. The Gini coefficient is computed as follows:

**Step 1**  To determine the number of people in an interval dy, we use the following expression:
  (7) dn=Pdy,
  where *P* represents the total number of individuals in the population.
**Step 2**  Next, to calculate the total amount of money accumulated by these individuals, we use
  (8) dz=xdn=Q(y)Pdy.
**Step 3**  The total amount of money accumulated by society can be calculated using the following expression:
  (9) $$Z=P\int_{0}^{1}Q\left(y^{\prime }\right)dy^{\prime }.$$
**Step 4**  The total amount of money accumulated by a fraction *y* of the population is given by
  (10) $$z\left(y\right)=P\int_{0}^{y}Q\left(y^{\prime }\right)dy^{\prime }$$
**Step 5**  The fraction of the total amount of money *Z* accumulated by a fraction *y* of the population, as represented by the Lorenz curve, is given by
  (11) $$z=L\left(y\right)=\frac{\int_{0}^{y}Q\left(y^{\prime }\right)dy^{\prime }}{\int_{0}^{1}Q\left(y^{\prime }\right)dy^{\prime }}.$$

In a perfectly equal society, the Lorenz curve is linear, represented by L(y)=y. This corresponds to Q(y)=1.

The Gini coefficient is calculated as follows:

(12) $$G=1-2\int_{0}^{1}L\left(y\right)dy.$$

Table 4 displays the Gini coefficients for the period 2012–2022, calculated using our metalogistic treatment alongside those reported by the World Bank [41]. There is a notable agreement between the two sets of results, with a root mean square deviation of 0.009, approximately 1% of the average Gini coefficient over the eleven years. The highest deviation, 3.6%, occurred in 2021, one year after the COVID-19 pandemic. The alignment of our results with those in Ref. [41] confirms the accuracy and reliability of our methodology.

### 3.3. Inflection Points of the Metalogistic Probability Density Function

**Figure 4:**
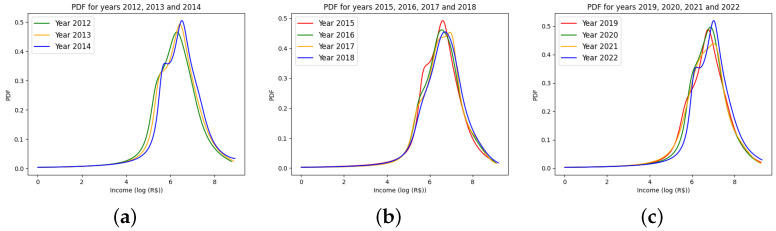
Metalogistic PDF for the presidential terms: the last three years of 2011–2014 (**a**), 2015–2018 (**b**), and 2019–2022 (**c**).

We organized the metalogistic probability density functions (PDFs) according to their respective presidential terms. Figure 4a illustrates the data for the period from 2012 to 2014. The plots reveal a concentration of income at higher values, as indicated by the widths and medians of the distributions.

Figure 4b presents the PDFs for the subsequent term, where no significant differences in medians and widths are observed. However, there is a noticeable shift in the bump on the left side of the PDF, which signifies the gradual disappearance of a particular income class during this period.

It is important to note that the subsequent period, 2019–2022, coincided with and followed the COVID-19 pandemic. Figure 4c shows a concentration of income shifting to higher values, reflecting a society in the process of recovery. Additionally, the reappearance of bumps on the left side of the distribution suggests the emergence of a new income class during this period.

**Figure 5:**
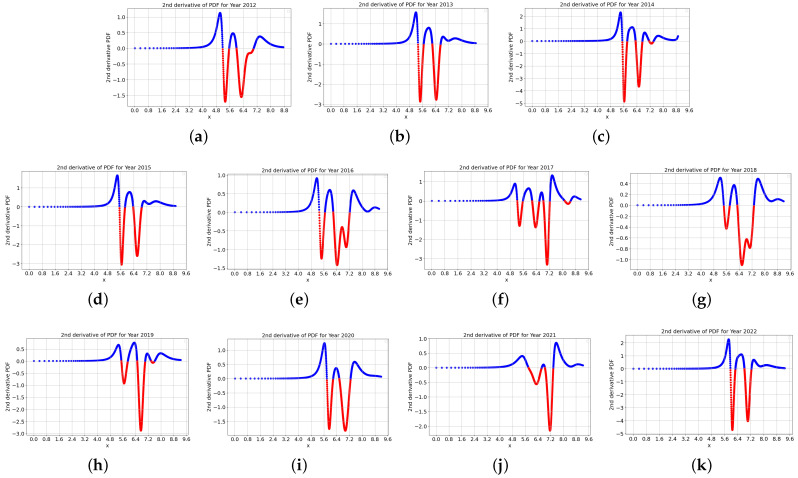
Second derivative of the PDF through the years 2012 (**a**) up to 2022 (**k**). The ascending PDF rates are represented in blue, while the descending rates are in red. The points where the curves change colors show the inflection points that define the boundaries of classes in our model.

Figure 5 display the second derivative of the probability density functions (PDFs) for each year. Ascending rates are represented in blue, while descending rates are shown in red. The points where the color changes indicate inflection points, which define the boundaries of income classes in our model.The plots for 2012 (a), 2013 (b), and 2014 (c), corresponding to the first presidential term, exhibit similar patterns. Although the general pattern persisted in 2015 (d), significant changes are evident from 2016 (e) to 2018 (g). This period was marked by events such as the impeachment of the Brazilian President in 2016–2017 and the global impact of COVID-19 in 2019 (h). From 2020 (i) to 2022 (k), the income distribution shifts back to the patterns observed in 2012–2014, with a notable concentration of income at higher values.

## 4. Discussion

Table 5 and Table 6 present the boundary values for different income classes. Based on the inflection point criterion, we identify five primary classes. Notably, in 2014, Class V is subdivided into three distinct classes. By 2015, the distribution returns to five classes, with only minimal adjustments to the lower and upper boundaries.

A notable split in Class V occurs in 2017, which also affects Class IV. By 2018, the distribution returns to its previous configuration, with overall improvements in income levels. The data from 2019 reveal another split.

Overall, the data depict a society distributed across five income classes, with noticeable fluctuations and significant gains observed in each class over the period. Specifically, the gains are 217% for Class I, 202% for Class II, 227% for Class III, 127% for Class IV, and 170% for Class V.

Once the class boundaries are established, we use the quantile probability function (QPF) to determine the population fraction within each class. The results are detailed in Table 7 and Table 8. Over the eleven-year period, we observe significant shifts among the classes, indicating notable inter-class migrations. Specifically, between 2012 and 2013, there was a migration from Class IV downward to Class III and upward to Class V. In 2015–2016, we saw an upward movement from Classes I, II, and III, alongside a downward shift from Class V to Class IV. This was followed by a gradual return to the distribution pattern observed in 2013 by the end of the period.

**Figure 6:**
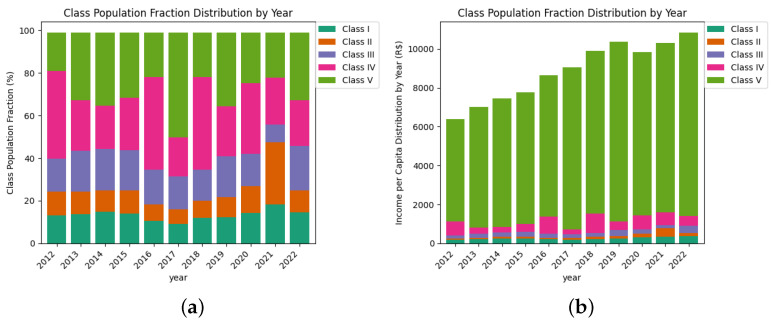
The bar plots illustrate the distribution of the population across different income classes (**a**) and the income thresholds defining each class (**b**) from 2012 to 2022.

The bar plots in Figure 6a,b provide a clearer visualization of the data from Table 5, Table 6, Table 7 and Table 8. They illustrate how the class boundaries fluctuate over time and help to infer migration patterns across these boundaries, as discussed earlier.

## 5. Conclusions

We developed a protocol for classifying distinct income categories within a society by using the inflection points of the probability density function of *per capita* income to define class boundaries. Two key observations are noteworthy:

- By using individual income instead of family income, we avoid distortions related to the fact that poorer families tend to be larger than wealthier ones. This discrepancy arises from both the higher number of children in poorer families and the broader definition of “family” in these households compared to wealthier ones.
- The boundaries between income classes vary annually rather than being fixed at predetermined levels. This variability is illustrated in Table 7, which shows how the distribution of the population across income classes evolves over time, reflecting the dynamic nature of societal income distribution.

To compare these classifications with those of IBGE, we need to map the class divisions from Table 5. For simplicity, we assume a family composition of two members for Classes A and B, three members for Class C, and four members for Classes D and E. Converting the income values to *per capita* figures reveals that IBGE’s Class E encompasses our Classes I, II, and III. This more detailed subdivision of Class E is considered advantageous for targeting social programs, aligning with Brazil’s state policies and informal job market dynamics. At higher income levels, IBGE’s Class C corresponds closely to our Class IV and the lower part of Class V, while IBGE’s Class B includes the upper portions of both Class IV and Class V. The top 1% not represented in IBGE’s Table 1 corresponds to Class A, which represents the wealthiest segment of the Brazilian population.

We can conclude that the criterion for class identification has proven consistent throughout the period. Applying our classification criterion—based on the zeroes of the second derivative of the probability density function—to the income distribution case study in Brazil shows that the resulting class divisions accurately reflect the dynamics of Brazilian society over the analyzed period. These findings suggest that our approach is a robust statistical method for understanding social dynamics and could be effectively applied to various other studies and surveys. 

## Figures and Tables

**Table 1:** Distribution of monthly *per capita* income (in Brazilian currency) in 2012–2022, according to Ref. [40]. The first column shows the interval as a percentage of the total population, while the subsequent columns display the upper limit of the percentile income for each year of the survey.

Percentile (%)	2012	2013	2014	2015	2016	2017	2018	2019	2020	2021	2022
<10	147	167	188	198	197	199	201	211	252	203	298
10–20	229	257	292	306	307	316	334	348	396	356	461
20–30	311	347	387	411	433	451	473	498	527	496	612
30–40	403	452	500	529	552	578	610	650	673	636	798
40–50	507	569	634	672	696	730	773	826	836	813	997
50–60	630	694	756	814	875	933	967	1002	1035	1053	1211
60–70	797	879	976	1029	1082	1130	1211	1282	1263	1273	1506
70–80	1074	1187	1300	1380	1479	1511	1641	1733	1682	1729	2022
80–90	1704	1907	2051	2177	2382	2453	2625	2723	2646	2742	3207
90–95	2688	2990	3236	3353	3714	3821	4067	4209	4082	4297	4948
95–99	6384	7021	7468	7758	8658	9049	9893	10365	9832	10,311	10,853

**Table 2:** Coefficients of the μ and *s* expansion in the metalog distribution.

Order	$a_{1}$	$a_{2}$	$a_{3}$	$a_{4}$	$a_{5}$	$a_{6}$	$a_{7}$	$a_{8}$	$a_{9}$	$a_{10}$
k=5	6.51	1.45	−2.00	−4.47	10.56					
k=6	6.49	2.85	−2.07	−9.16	11.39	−3.67				
k=7	6.50	−1.27	−2.05	7.66	11.10	10.44	−41.57			
k=8	6.59	6.09	−6.87	−22.13	25.70	−14.37	27.60	13.416		
k=9	6.58	8.05	−15.13	−30.06	59.85	−20.97	46.00	41.79	−85.41	
k=10	6.58	8.58	−15.35	−32.16	60.75	−23.88	52.71	42.539	−87.58	1.803

**Table 3:** Shannon entropy obtained with the the metalog distribution in Equation (3) with k=10 fitting the data in Table 1.

Year	2012	2013	2014	2015	2016	2017	2018	2019	2020	2021	2022
Shannon entropy	7.27	6.66	6.04	5.89	8.08	5.68	5.92	4.81	5.04	5.05	4.13

**Table 4:** Gini coefficients calculated after the PDF obtained via the metalogistic distribution from Table 1.

Year	Gini (Our Calculation)	Gini (World Bank)
2012	0.535	0.526
2013	0.533	0.521
2014	0.518	0.518
2015	0.523	0.522
2016	0.531	0.524
2017	0.522	0.524
2018	0.536	0.526
2019	0.540	0.543
2020	0.517	0.524
2021	0.539	0.520
2022	0.512	0.523

**Table 5:** Upper boundaries of the five income classes (in BRL) for 2012 and 2022, determined using the inflection points of the second derivatives of the PDF.

Classes	2012	2013	2014	2015	2016	2017	2018	2019	2020	2021	2022
ClassI	178	206	247	250	202	188	229	248	325	334	387
ClassII	261	292	334	354	289	266	334	372	483	768	527
ClassIII	402	492	556	582	486	467	536	672	708	938	914
ClassIV	1104	820	847	985	1368	729	1529	1112	1444	1593	1406
ClassV	6384	7021	7468	7758	8658	9049	9893	10,365	9832	10,311	10,853

**Table 6:** Upper boundaries of the Class V subdivisions (in BRL) for 2014, 2017, and 2019, as detailed in Table 5.

Classes	2012	2013	2014	2015	2016	2017	2018	2019	2020	2021	2022
ClassVa	-	-	1279	-	-	930	-	1615	-	-	-
ClassVb	-	-	1679	-	-	1377	-	2023	-	-	-
ClassVc	-	-	7468	-	-	3434	-	10,365	-	-	-
ClassVd	-	-	-	-	-	4925	-	-	-	-	-
ClassVd	-	-	-	-	-	9049	-	-	-	-	-

**Table 7:** Percentage of the population in each class for 2012–2022, calculated by integrating the PDF within each interval specified in Table 5. For Class V, the values represent the sum of its subdivisions as detailed in Table 8.

Class	2012	2013	2014	2015	2016	2017	2018	2019	2020	2021	2022
*I*	13.22	13.62	14.78	14.08	10.41	9.13	11.89	12.37	14.35	18.35	14.57
II	10.94	10.54	10.06	10.82	7.95	6.78	8.09	9.37	12.50	29.03	10.16
III	15.62	19.32	19.52	18.87	16.08	15.53	14.64	19.26	15.28	8.45	21.13
IV	41.05	23.82	20.25	24.52	43.47	18.32	43.40	23.35	32.92	21.96	21.24
*V*	18.17	31.70	34.38	30.70	21.08	49.25	20.97	34.65	23.95	21.21	31.89

**Table 8:** Population distribution inside the divisions of Class V in 2014, 2017, 2019, according to Table 7.

Class	2012	2013	2014	2015	2016	2017	2018	2019	2020	2021	2022
Va	-	-	14.78	-	-	10.74	-	13.57	-	-	-
Va	-	-	6.94	-	-	16.81	-	6.17	-	-	-
Vb	-	-	12.66	-	-	16.67	-	14.91	-	-	-
Vc	-	-	-	-	-	3.02	-	-	-	-	-
Vd	-	-	-	-	-	2.01	-	-	-	-	-

## Data Availability

All data are available upon request.
